# 免疫检查点抑制剂相关毒副作用管理之激素的使用

**DOI:** 10.3779/j.issn.1009-3419.2019.10.02

**Published:** 2019-10-20

**Authors:** 汉萍 王, 佳鑫 周, 潇潇 郭, 玥 李, 炼 段, 晓燕 斯, 力 张

**Affiliations:** 1 100730 北京，中国医学科学院，北京协和医院呼吸内科 Department of Respirology, Peking Union Medical College Hospital, Chinese Academy of Medical Sciences, Beijing 100730, China; 2 100730 北京，中国医学科学院，北京协和医院风湿免疫科 Department of Rheumatology and Immunology, Peking Union Medical College Hospital, Chinese Academy of Medical Sciences, Beijing 100730, China; 3 100730 北京，中国医学科学院，北京协和医院心内科 Department of Cardiology, Peking Union Medical College Hospital, Chinese Academy of Medical Sciences, Beijing 100730, China; 4 100730 北京，中国医学科学院，北京协和医院消化内科 Department of Gastroenterology, Peking Union Medical College Hospital, Chinese Academy of Medical Sciences, Beijing 100730, China; 5 100730 北京，中国医学科学院，北京协和医院内分泌科 Department of Endocrinology, Peking Union Medical College Hospital, Chinese Academy of Medical Sciences, Beijing 100730, China

**Keywords:** 免疫检查点抑制剂, 免疫相关不良反应, 糖皮质激素, 免疫抑制, Immune checkpoint inhibitor, Immunotherapy-related adverse effects, Glucocorticoid, Immune suppressors

## Abstract

免疫检查点抑制剂（immune checkpoint inhibitors, ICIs）通过激活机体的肿瘤免疫杀灭肿瘤细胞，给肿瘤患者的治疗带来新的希望。然而也可引起一系列以免疫损伤为基础的免疫检查点抑制剂相关毒副反应（immune-related adverse events, irAEs）。糖皮质激素是治疗这类毒副反应的基础，而在具体治疗irAEs时，其用法、用量、疗程又与治疗经典的自身免疫性疾病有所不同。同时，大剂量的糖皮质激素的长期使用可能引起严重的不良反应，本文对激素的作用机制、剂量剂型、毒副作用及管理等方面进行详细描述，为肿瘤医生在临床实践中应用激素提供参考和建议。

## Abstract (en)

Immunocheckpoint inhibitors (ICIs) activated the patients' tumor immunity to kill the tumor cell, and brought new hope to patients with tumor. However, a series of immunocheckpoint inhibitors related adverse effects (irAEs) may also occur based on immune injury. Glucocorticoids are the basis for the treatment of such irAEs. However, the usage, dosage and course of treatment of glucocorticoid in irAEs are different from those in classic autoimmune diseases. Meanwhile, long-term use of large doses of glucocorticoids may cause serious adverse effects too. In this paper, the mechanism, dosage forms, adverse effects and management of glucocorticoids are described in detail, providing references and suggestions for oncologists to apply glucocorticoids in clinical practice.

免疫检查点抑制剂（immune checkpoint inhibitors, ICIs）通过特异性结合到相应的免疫检查点上，将免疫检查点的抑制作用取消，使得机体的肿瘤免疫得到激活，达到杀灭肿瘤细胞的作用。然而，激活的免疫系统也可能会攻击人体正常的器官脏器，引起相关的免疫毒副反应，这种免疫损伤可能累及身体的各个部位，引起相应的症状，成为一类新的病因明确的免疫系统异常性疾病，称之为免疫检查点抑制剂相关毒副反应（immunocheckpoint inhibitors related adverse effects, irAEs）^[1]^。由于其免疫损伤及炎症反应的本质，治疗这类疾病的基本策略就是通过使用糖皮质激素和免疫抑制剂来抑制这种被ICIs异常激活的免疫和炎症。糖皮质激素是一类具有强大且快速的抑制炎症和免疫反应能力的药物。而激素在治疗irAEs这类特殊类型的免疫性疾病时，其用法、用量、疗程又与治疗经典的自身免疫性疾病有所不同。

20世纪30年代不同研究小组的成员分别先后分离出了糖皮质激素皮质醇的非活性形式可的松（cortisone），并实现了人工合成皮质醇。此后10年，糖皮质激素逐渐应用于临床。1950年-1960年间新合成的糖皮质激素强的松及甲基强的松龙具有更强大的抗炎和免疫抑制作用，而盐皮质激素方面的副作用大大减少，因此得到了更加广泛的应用^[2]^。尽管如此，长期使用大剂量的糖皮质激素仍有可能会引起严重的不良反应，如对内分泌、骨骼、皮肤、肌肉、眼睛等的副作用，以及机会性感染等，如处理不当经常会导致严重不良后果。因此，本文将对激素的作用机制、剂量剂型、毒副作用及管理等方面进行详细描述，为肿瘤医生在临床实践中应用激素提供参考和建议。

## 1 糖皮质激素的分子作用机制

糖皮质激素作用的不同分子机制为优化糖皮质激素的使用提供了依据。研究表明，糖皮质激素的分子作用机制可以分为基因组效应和非基因组效应^[3, 4]^。其中经典的基因组效应为细胞浆糖皮质激素受体alpha（cytosolic glucocorticoid receptor alpha, cGCR）介导，非基因组糖皮质激素活性可进一步分为三种作用模式：cGCR介导的非基因组效应；非特异性非基因组效应（例如，高糖皮质激素浓度下与质膜的物理化学相互作用）；以及被认为是由膜结合糖皮质激素受体介导的效应^[4]^。

### 1.1 经典的cGCR介导的基因组效应

细胞浆内存在cGCR，这是一个多蛋白复合的分子，包含几个热休克蛋白（heat shock protein, Hsp），如Hsp90、Hsp70、Hsp56以及Hsp40等，也可和免疫亲和蛋白、伴侣蛋白（如p23、Src），及线粒体激活蛋白激酶（mitogen-activated protein kinase, MAPK）信号通路激酶相互作用。糖皮质激素为脂溶性，容易透过细胞膜进入细胞内，与cGCR结合后形成激活的GC/cGCR复合体，这时cGCR上连接的伴侣蛋白就会游离到细胞浆内。随后GC/cGCR复合物会在20 min内转移进入细胞核，通过与特定的DNA结合位点结合，影响细胞核内的转录和翻译过程，上调免疫抗炎蛋白的合成，下调炎症介质的释放，起到免疫抑制的效果。这种调节蛋白浓度的显著变化需要30 min以上的时间。而从激活cGCR、受体复合物的核内转运、与靶基因启动子区域的GREs结合，以及转录和翻译的启动，直至产生新的蛋白质，通常需数小时或数天^[3]^。因此激素更快的抗炎、抗过敏的疗效不能完全用经典的基因组效应来解释。

### 1.2 激素的快速免疫抑制效应

三种不同的非基因组机制或可解释GC的快速抗炎和免疫抑制作用^[5]^：（1）糖皮质激素与细胞膜的非特异性相互作用：大剂量GC可直接穿透细胞膜，改变了膜相关蛋白的理化性质和活性，导致钙和钠在免疫细胞细胞膜上的循环减少，有助于快速的免疫抑制和炎症抑制。（2）由胞质GR（cytosolic glucocorticoid receptor, cGCR）介导的非基因组效应：如前所述，未结合的GCR是一个多蛋白复合物，其与GC结合后，配体如Src、热休克蛋白及信号分子从GCR-多蛋白复合物中释放出来，产生快速的GC效应。（3）膜结合GCR（membrane-bound GCR, mGCR）介导的非基因组效应：在免疫细胞的表面有膜结合GCR的存在，GC与mGCR结合后，可快速产生mGCR介导的非基因组效应。

## 2 激素对免疫系统的作用

激素对于多种初级及次级炎症细胞都具有作用，具体见表 1^[6]^。

**1:** 糖皮质激素对原发和继发免疫细胞的影响 Effects of glucocorticoids on primary and secondary immune cells^[6]^

Immune cell type	Effection
Macrophages and Monocytes	Decrease number of circulating cells Decrease expression of MHC class Ⅱ molecules and Fc receptors Decrease synthesis of pro-inflammatory cytokines (*e.g*. IL-2, IL-6, TNF) and prostaglandins
Lymphocyte	Decrease number of circulating cells Decrease proliferation and impaired activation Decrease production and action of IL-2 (most important) (Little effect on the secretion of immunoglobulin)
Granulocytes	Decrease number of eosinophile and basophile granulocytes Increase number of circulating neutrophils
Endothelial cells	Decrease vessel permeability, decrease expression of adhesion molecules and production of IL-1 and prostaglandins
Fibroblasts	Decrease proliferation, decrease production of fibronectin and prostaglandins
IL-2: interleukin 2; IL-6: interleukin 6; TNF: tumor necrosis factor.	

糖皮质激素降低多种炎症细胞的活化、增殖、分化和存活，包括T淋巴细胞和巨噬细胞，通过对细胞因子的产生和分泌的调节，促进细胞的凋亡，尤其是未成熟和活化的T细胞的凋亡。而B淋巴细胞和中性粒细胞对于糖皮质激素的敏感性较低，因此很少影响免疫球蛋白的产生。激素可轻微刺激中性粒细胞，致其数量增加。另外，糖皮质激素可抑制黏附分子的减少，抑制内皮细胞的黏附，减少血管通透性，减少炎性渗出。超生理浓度的糖皮质激素还可以抑制成纤维细胞增殖、白介素1（interleukin 1, IL-1）和肿瘤坏死因子α（tumor necrosis factor α, TNFα）的分泌。通过这些作用，激素可以产生有效的抗炎作用。目前认为ICIs治疗导致人体T细胞肿瘤免疫的异常激活，由此引起的毒副作用主要为T细胞淋巴细胞的炎症，在多种类型irAEs的病理活检结果中都得到了证实（如心肌活检、肺活检、皮肤活检等）^[7-9]^，因此激素是治疗这类特殊毒副作用的最重要用药^[10]^。

## 3 激素的种类

临床常用的激素种类见表 2^[6]^。

**2:** 激素种类及其药动学特征 Types of glucocorticoids and their pharmacokinetic characteristics

		Equivalent dose (mg)	Relative glucocorticoid activity	Relative mineralocorticoid activity	Protein binding capacity	plasma half-life (h)	Biological half-life (h)
Short-acting	Cortisone	25	0.8	0.8	-	0.5	8-12
	Hydrocortisone	20	1	1	++++	1.5-2	8-12
Intermediate-acting	Methylprednisolone	4	5	0.5	-	> 3.5	18-36
	Prednisolone	5	4	0.6	++	2.1-3.5	18-36
	Prednisone	5	4	0.6	+++	3.4-3.8	18-36
	Triamcinolone	4	5	0	++	2- > 5	18-36
Long-acting	Dexamethasone	0.75	20-30	0	++	3-4.5	36-54
	Betamethasone	0.60	20-30	0	++	3-5	36-54
-: No；++: High; +++: High to very high; +++: very high.							

根据糖皮质激素的药代动力学，按照半衰期长短，糖皮质激素可以分为短效、中效及长效激素。大多数口服糖皮质激素吸收很快，30 min即可吸收，强的松和强的松龙的口服生物利用度高。血浆中90%-95%的皮质醇与血浆蛋白结合，不具有生物学活性，剩下5%-10%的游离皮质醇具有生物活性。因此，血浆白蛋白水平会影响激素的疗效及副作用，对于低蛋白血症、肝病的患者，应考虑调整剂量。

处理3级-4级irAEs时，激素的起始剂量多需1 mg/kg-2 mg/kg或以上的泼尼松，总疗程多在4周-6周或以上^[10, 11]^，为更好的控制剂量及毒副作用，同时尽量减少激素的副作用及对肾上腺垂体轴的生理抑制，多选择中效激素，例如泼尼松或甲基泼尼松龙。地塞米松的抗炎作用较强，但是生物半衰期长，长期使用时副作用更加明显，故一般不选择用地塞米松来控制irAEs。

## 4 激素的临床应用原则

如前所述，由于激素具有基因组效应和非基因组效应两类作用机制，不同剂量的激素可以带来不同强度和不同速度的的效力及毒副作用。在风湿免疫性疾病的治疗中，临床上根据疾病损伤的靶器官、疾病严重程度、疾病炎症发展的速度及潜在的危及生命的风险，来选择不同剂量的激素进行治疗。激素的剂量等级及其在风湿性疾病中的临床应用见表 3。

**3:** 风湿病激素剂量水平分类 Classification of dose levels of glucocorticoids in rheumatological diseases

Classification	Equivalent dose of prednisone	Clinical application	Genomic actions (receptor saturation)	Nongenomic actions	Adverse effects
Low dose	≤7.5 mg/d	Maintenance therapy for many rheumatic diseases	+ (< 50%)	？	Relatively few
Mediun dose	> 7.5 mg/d to ≤30 mg/d	Initial treatment for primary chronic rheumatic diseases	++ (> 50% to < 100%)	+	Dose-dependent, more if used for a long time
High dose	> 30 mg/d to ≤100 mg/d	Initial treatment for subacute rheumatic diseases	++ (almost 100%)	+	Cannot be used for long term because of serious adverse effects
Very high dose	> 100 mg/d	Initial treatment for acute and/or potentially life-threatening exacerbations of rheumatic diseases	+++ (almost 100%)	++	Cannot be used for long term because of very serious adverse effects
Pulse therapy	≥250 mg/d for one or few days	For particularly severe and/or potentially lifethreatening forms of rheumatic diseases	+++ (100%)	+++	More early and serious adverse effects, and more unusual adverse effects

大剂量糖皮质激素同时具有基因组效应和非基因组效应，适用于起病较急病情较严重的风湿性疾病。而激素的剂量越大，基因组效应和非基因组效应都越强，可起到更快更强的免疫抑制作用，因此在危及生命的风湿性疾病中，常考虑采用超大剂量或冲击剂量的激素进行治疗。然而，更大剂量的激素，也会带来更多的副作用，尤其在冲击剂量激素，经常会带来一些其他剂量激素应用时不常见的少见副作用，因此，超大剂量或冲击量的激素应用时间往往较短，以尽可能地控制毒副作用。

对于明确诊断irAEs的患者，早期加用糖皮质激素是关键。在具体使用原则上，可借鉴风湿性疾病中激素的应用原则，通常按照受累脏器，严重程度、病程缓急等因素考虑激素用量（见表 4）^[11]^。对于病程紧急、容易危及生命的重大脏器损伤，如心肌毒性、神经毒性，尤其是暴发性心肌炎或横贯性脊髓炎患者，往往病程急、进展快，死亡率高，推荐冲击量激素，以最大程度且最快速地阻断免疫损伤。对于其他脏器的3级-4级AE，如消化道、皮肤、肺部、肾脏等脏器的损伤，推荐大剂量的激素治疗（泼尼松1 mg/kg/d-2 mg/kg/d或其等效剂量），兼顾疗效和副作用，而对于内分泌系统的irAEs，免疫损伤多以抗体介导为主，很少存在全身的炎症反应，损伤后主要以内分泌腺的分泌不足为主要表现，则以补充生理剂量的激素为主。剂型方面，胃肠道损伤者影响口服药物的吸收，建议以静脉激素治疗为主，对于皮肤损伤的患者，可合并使用外用的皮质类固醇药物进行治疗。

**4:** NCCN指南推荐的激素使用原则 Principles for the use of glucocorticoids recommended by NCCN guidelines

irAEs	Grade	Type of hormne	Initial dose（mg/kg/d）
Dermatologic	G2 G3-4	Prednisone Prednisone/Methylprednisolone	0.5-1 0.5-1-2
Diarhea/Colitis	G2 G3-4	Prednisone/Methylprednisolone Methylprednisolone	1 2
Hepatic toxicity	G2 G3 G4	Prednisone Prednisone/Methylprednisolone Methylprednisolone	0.5-1 1-2 2
Pancreatitis	G2 G3-4	Prednisone/Methylprednisolone Prednisone/Methylprednisolone	0.5-1 1-2
Pulmonary	G2 G3-4	Prednisone/Methylprednisolone Prednisone/Methylprednisolone	1-2 1-2
Renal	G2 G3-4	Prednisone Prednisone/Methylprednisolone	1-2 1-2
Musculoskeletal	G2 G3-4	Prednisone	0.5 1
Guillain-Barré syndrome Transverse myelitis	G2/G3/G4	Methylprednisolone	1, 000 mg/d
Cardiovascular	G3 G4	Methylprednisolone	1, 000 mg/d
NCCN: National Comprehensive Cancer Network.			

疗程方面，一般在4周以上，有时6周-8周或更长，尤其对于易复发的肺炎和肝炎。

不推荐常规预防性使用激素。

## 5 激素的副作用及管理

长期的激素治疗会带来一系列的毒副作用，累及全身多个系统，包括：骨骼肌肉副作用，如骨质疏松、骨坏死、类固醇肌病，消化系统，如消化性溃疡、出血、胰腺炎、脂肪肝，心血管系统方面，包括高血压、早发动脉硬化、心律失常；代谢内分泌副作用，如糖、脂代谢紊乱、水钠储留、电解质紊乱、HPA轴抑制、性腺抑制、食欲和体质量增加；精神行为方面，如失眠、情绪不稳、认知障碍；合并机会性感染（包括真菌、TB、PCP等）；白内障、青光眼，以及皮肤痤疮、紫纹、皮肤变脆、瘀斑、多毛、伤口不愈合等^[6]^。

然而，患者出现各种副作用的风险与激素的剂量和疗程呈正相关，其中低剂量激素感染风险明显降低，但是合并其他基础疾病且未控制者感染风险也可增加，如糖尿病血糖未控制者、低蛋白血症未纠正者等。临床上，各种副作用的出现时间各不相同。患者第一次使用激素后，即有可能出现精神症状，1天-3天起可表现出水钠潴留、电解质紊乱、心率增快、血压升高等情况，1周左右可能出现血糖的升高，机会性感染一般发生在激素治疗3周及以后，而激素冲击治疗后则感染的发生可明显提前。长期使用激素超过2个月者要注意骨质疏松的发生，出现Cushing面容者一般激素时间在3个月以上，而出现肾上腺皮质功能抑制者激素的使用时间更久。

因此，针对irAEs的激素使用原则，首先应该从合适的剂量起始，大剂量的激素时间最好控制在2周-3周之内，待疾病控制后注意及时减量，用药期间注意检测血压、血糖、电解质、出入量等，警惕感染。

预防性用药方面，在大剂量激素，尤其是冲击量激素期间或者合并消化道出血高危因素的患者，考虑加用质子泵抑制剂治疗或H2阻滞剂；对于强的松≥20 mg/d，持续4周或4周以上者，可考虑预防PCP；常规补充维生素D和钙^[11]^。另外，激素使用期间，应注意患者的宣教，避免感染或避免接触感染源，注意饮食控制，避免大幅度体重增加，监测血糖等^[10]^。

激素使用后发生感染的风险及机会感染的病原学与血液中CD4^+^ T淋巴细胞计数减少明显相关，同时与合并的血液情况，患者肺部情况等相关。临床上应注意观察激素治疗期间的病情变化，警惕相应的副作用尤其是感染。对于合并感染者则应早期经验性抗生素治疗，根据患者不同的免疫抑制水平覆盖特殊病原体，并积极寻找病原学证据调整治疗。
